# Comorbidities and their management in women with breast cancer—an Australian survey of breast cancer survivors

**DOI:** 10.1007/s00520-023-07678-7

**Published:** 2023-03-14

**Authors:** Bogda Koczwara, Rosie Meng, Malcolm Battersby, Arduino A. Mangoni, Danielle Spence, Sharon Lawn

**Affiliations:** 1 College of Medicine and Public Health, Flinders University, Adelaide, Australia; 2 Cancer Council Victoria, Melbourne, Australia

**Keywords:** Chronic disease, Comorbidity, Breast cancer

## Abstract

**Purpose:**

Breast cancer survivors experience significant burden from comorbid chronic conditions, but little is known about how well these conditions are managed. We conducted a national survey of Australian breast cancer survivors to examine the burden of chronic conditions, their impact and care alignment with the principles of chronic condition management.

**Methods:**

A study-specific survey incorporated questions about chronic conditions using the Charlson Comorbidity Index (CCI), functional status using the Vulnerable Elders Survey (VES) and perceived quality of care for cancer and non-cancer conditions using the Patient Assessment of Care for Chronic Conditions Survey (PACIC). Members of Breast Cancer Network Australia (BCNA) were invited via email to complete the survey either online or through direct mail.

**Results:**

The survey was sent to 2198 BCNA members and 177 responses were received (8.1%). Respondents were women aged 32–88 years (median 60.1 years). The majority were married (116; 67.7%) and had private insurance (137; 80.0%) and reported good to excellent health (119; 73.5%). Other health conditions were reported by 157 (88.7%), the most common being chronic pain (27.1%) and fatigue (22.0%). When asked about management of comorbidities or cancer, less than 20% were routinely asked about management goals, helped to set goals or asked about health habits.

**Conclusions:**

In this population of survivors with good health status and high rates of private insurance, comorbidities were common and their management, as well as management of breast cancer, was poorly aligned with chronic condition management principles.

**Supplementary Information:**

The online version contains supplementary material available at 10.1007/s00520-023-07678-7.

## Introduction

Comorbidities are common in patients with breast cancer, especially those who are older, because of their increasing prevalence with advancing age and shared risk factors for cancer and many chronic conditions [1]. Furthermore, increasingly evidence suggests that survivors of breast cancer are at higher risk of developing new chronic conditions compared to cancer-free controls [2, 3] although this pattern has not been replicated in all studies [4]. For example, in a study of over 900 breast cancer patients in the USA, 66% of white and 86% of black patients had at least one comorbidity and 28% and 35% respectively had 3 or more [5]. Similarly, a report from McMillan Cancer Charities in the UK showed that four out of five women who were 7 years or more post completion of treatment for breast cancer had comorbidities that required inpatient management [6]. The report, if anything, likely underestimated the rates of comorbidities as it focused only on those severe enough to require hospitalisation.

The presence of comorbidities has been shown to influence treatment choice, uptake and toxicity, cancer and non-cancer survival, quality of life and cost of care, making it a priority for research and practice in cancer [7–10]. Indeed, the management of comorbid conditions is explicitly recognised as an important part of effective survivorship care [11] but the delivery of effective care of comorbid chronic conditions in the context of cancer poses several potential challenges. For example, the management of comorbid conditions requires greater care coordination within the cancer setting and the broader health care setting with input from other health care professionals, especially primary care providers who have the necessary skills to manage chronic conditions [12]. The care of comorbidities may not be prioritised by the patient or their health care providers [13]. There are limited tools and care pathways that explicitly integrate management into the breast care pathway [14]. Lastly, there is a relative scarcity of evidence regarding the management of comorbidities. A recent umbrella review of reviews related to interventions for breast cancer survivors identified that out of 323 reviews only seven (2%) addressed the management of chronic conditions [15]. A qualitative systematic review of cancer and comorbid illness demonstrated relative scarcity of evidence on patient experiences of living with comorbid illness [16].

To better understand the pattern of comorbid conditions experienced by breast cancer survivors, their impact and the quality of their care, we conducted a survey of Australian cancer survivors using validated measures of comorbidity and chronic condition management. Specifically, the survey aimed to address the following objectives: (1) examine the self-reported prevalence of comorbidity in women with history of breast cancer; (2) evaluate the impact of comorbidities on self-perceived health status; and (3) assess the quality of care delivered for management of comorbid chronic conditions as compared to care delivered for cancer.

## Methods

A study-specific survey was developed and pilot tested with a small group of researchers and consumers. In addition to demographic questions, the survey incorporated questions about the presence of chronic conditions, functional status and perceived quality of care for cancer and non-cancer conditions (Supplementary material 1) . Comorbidity burden was assessed using the Charlson Comorbidity Index (CCI)—a validated measure that lists 23 chronic conditions plus an option of including “other” and directly indicating that condition [17]. The entries for “other” conditions were reviewed and if appropriate added to the main categories and the CCI score was calculated. Functional status was assessed using the Vulnerable Elders Survey (VES)—a validated 13-item function-based scoring system that considers age, self-rated health, limitation in physical function and functional disabilities [18]. The perceived quality of care for cancer and non-cancer conditions was assessed using the Patient Assessment of Care for Chronic Conditions Survey (PACIC) [19]. PACIC includes 20 questions across five subscales: patient activation; delivery system design/decision support; goal setting; problem-solving/contextual counselling; and follow-up/coordination. Study participants were asked to complete PACIC questions about the management of the conditions other than cancer and again about management of the cancer itself.

Members of Breast Cancer Network Australia (BCNA) were invited via email to complete the survey either online or they could request a hard copy of the survey to be posted and returned via mail. BCNA is a national advocacy organisation of approximately 100,000 members, many of whom have previously indicated willingness to take part in surveys relevant to breast cancer. Completion of the questionnaire implied consent. Ethical approval for the study was provided by the Southern Adelaide Local Health Network Hospital Research Ethics Committee (application 367.16).

Differences in PACIC score between chronic disease care and cancer care were assessed using paired-sample *t*-test and mixed effect model. The distribution of PACIC overall score and five subscale scores were assessed using histogram and normality test. None of these measures is normally distributed, and none of conventional transformation could achieve normal distribution. Therefore, the Wilcoxon matched-pairs signed-ranks test was also performed. The between group differences in PACIC were also assessed by using multivariable mixed effect model, in which patient’s demographic variables and VES score were included for adjustment. All analyses were performed using Stata MP 14.1 (StataCorp, TX, USA). All tests were two-sided, with a *p* value < 0.05 indicating statistical significance.

## Results

The survey was sent to 2198 members of BCNA directly from BCNA. The researchers had no direct contact with potential participants and only one invitation to complete the survey was sent. A total of 177 responses were received (response rate 8.1%) but not all respondents completed all questions. All were women with mean age of 60.1 years (range 32–88). The majority had Australian cultural background (85.9%), were married (67.7%), had private health insurance (80.0%), and approximately a third were employed (37.6%). The majority described their health as good, very good or excellent (73.5%) (Table 1).

**Table 1:** Demographic characteristics of respondents (*n* = 177)

	*n* (%)
Agree to proceed the interview	*n* = 177
Age in years, *n* = 149	60.1 (9.3)
Marital status, *n* = 170	
Single	21 (13.4)
Married	115 (67.7)
De facto	13 (7.6)
Divorced	13 (7.6)
Widowed	7 (4.1)
Prefer not to tell	1 (0.6)
Employment status, *n* = 170	
Unemployed	3 (1.8)
Employed	64 (37.6)
Retired	80 (47.1)
Home duties	11 (6.5)
Other	11 (6.5)
Prefer not to tell	1 (0.6)
Income, *n* = 170	
$0–$6000	3 (1.8)
$6000–$35,000	40 (23.5)
$35,000–$80,000	47 (27.7)
$80,000–$180,000	34 (20.0)
Over$180,000	13 (7.6)
Prefer not to tell	33 (19.4)
Living arrangement, *n* = 169	
Other living arrangement	134 (79.3)
Living alone	33 (19.5)
Prefer not to tell	2 (1.2)
Had private health insurance, *n* = 170	
No	34 (20.0)
Yes	136 (80.0)
Culture background, *n* = 170	
Other	24 (14.1)
Australia	146 (85.9)
General health, *n* = 162	
Poor	8 (4.9)
Fair	35 (21.6)
Good	73 (45.1)
Very good	44 (27.2)
Excellent	2 (1.2)
VES score, mean (SD), *n* = 141	2.7 (2.2)

Chronic conditions other than cancer were reported by 157 (88.7%) respondents. The median number of chronic conditions reported was three; with 40 women (22.7%) reporting four or more. The majority of respondents (63.8%) reported the presence of a condition that was not explicitly listed in the CCI. The most common comorbidities included chronic pain (27.1%), persistent fatigue (19.8%), chronic obstructive pulmonary disease (16%), osteoporosis (15.8%), peripheral neuropathy (15.2%) and arthritis (14.1%). Of these, only airways disease was explicitly included in the CCI scores—the remainder were classified as “other” (Table 2). Both the number of chronic conditions and the CCI score correlated with inferior perceived health (rho =  − 0.29, *p* < 0.001; and rho =  − 0.24, *p* = 0.002, respectively) and the VES score (rho = 0.37, *p* < 0.001; and rho = 0.23, *p* = 0.007, respectively).

**Table 2:** Comorbid chronic conditions

	*n* (%)
	(*n* = 177)
Presence of any chronic condition (CC)	157 (88.7)
Conditions listed in CCI	
Chronic obstructive pulmonary disease	29 (16.4)
Arm or leg weakness	24 (13.6)
Mental disorder (including depression and bipolar)	23 (13.0)
Diabetes with chronic complication	16 (9.0)
Renal disease	4 (2.3)
Peptic ulcer disease	1 (0.6)
Myocardial infarction	3 (1.7)
Congestive heart failure	5 (2.8)
Peripheral vascular disease	2 (1.1)
Cerebrovascular disease	1 (0.6)
Liver disease (mild)	7 (4.0)
Liver disease (moderate/severe)	2 (1.1)
Leukaemia	1 (0.6)
Malignant tumour—not metastatic	169 (95.5)
Malignant tumour—metastatic	8 (4.5)
Chronic conditions (other than those listed in CCI)	
Chronic pain	48 (27.1)
Persistent fatigue	35 (22.0)
Osteoporosis	28 (15.8)
Peripheral neuropathy	27 (15.2)
Arthritis	25 (14.1)
Lymphedema	19 (10.7)
Obesity	14 (7.9)
Total CCI score (age adjusted, mean (SD))	4.4 (1.9)
CC number (as used in CCI scoring)	
1	93 (52.5)
2	52 (29.4)
3	26 (14.7)
4	5 (2.8)
6	1 (0.6)
Total number of any CC	
1	34 (19.3)
2	50 (28.4)
3	52 (29.6)
4 or more	40 (22.7)

**Fig. 1:**
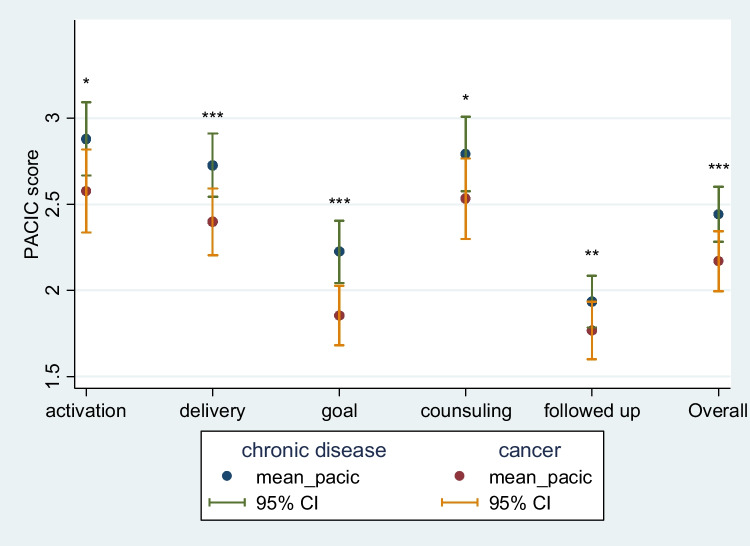
Overall PACIC scores and scores of five subscales for chronic disease and cancer care. *p < 0.05, **p < 0.01, ***p < 0.001, derived from paired sample *t*-test

When asked about management of chronic conditions, 49 (34%) respondents said they were never asked for input into their management plan, 29 (20%) were never given choices about treatment, 40 (28%) were never asked to talk about side effects of medicines, 49 (35%) were never advised about self-management options, 65 (45%) were never asked about goals of care, and 63 (44%) were never asked about health habits (Table 3). Corresponding rates for cancer care were 51 (40%) for never asked for input into management, 44 (34%) for never given choices about treatment, 44 (34%) for never being asked about side effects, 89 (69%) for never being advised about self-management options, 72 (56%) for never being asked about goals of care and 73 (57%) for never being asked about health habits (Table 4). Overall, 48 (35%) were never asked how their chronic condition affected their life and 84 (61%) were never encouraged to attend community programs to help with the management of chronic conditions. Corresponding figures for cancer were 58 (46%) and 89 (70%), respectively. Overall, mean PACIC overall score and five subscale scores, whilst generally low, were higher for management of chronic conditions compared to cancer care management, and these results were confirmed by the rank test results (Fig. 1, Table 5, all *p* < 0.05 in *t*-test). The significance remained when adjusted for demographic variables (*p* < 0.05).

**Table 3:** Response to the request to complete the statement: “*Over the past 6 months, when I received care for my chronic conditions aside from cancer, I was…*”

	None of the time (*n*; %)	A little of the time (*n*; %)	Some of the time (*n*; %)	Most of the time (*n*; %)	Always (*n*; %)	Total (*n*)
Asked for my ideas when we made a treatment plan	49; 34.0%	17; 11.8%	31; 21.5%	22; 15.3%	25; 17.4%	144
Given choices about treatment to think about	29; 20.3%	23; 16.1%	33; 23.1%	28; 19.6%	30; 21.0%	143
Asked to talk about any problems with my medicines or their effects	40; 28.0%	18; 12.6%	29; 20.3%	28; 19.6%	28; 19.6%;	143
Given a written list of things I should do to improve my health	75; 53.6%	20; 14.23%	16; 11.4%	14; 10.0%	15; 10.7%	140
Satisfied that my care was well organized	12; 8.5%	23; 16.2%	25; 17.6%	50; 35.2%	32; 22.5%	142
Shown how what I did to take care of myself influenced my condition	49; 35.0%	23; 16.4%	24; 17.1%	26; 18.6%	18; 12.9%	139
Asked about my goals in caring for my condition	65; 45.8%	25; 17.6%	22; 15.5%	19; 13.4%	11; 7.78%	138
Helped to set specific goals to improve my eating and exercise	65; 46.8%	23; 16.6%	27; 19.4%	16; 11.5%	8; 5.%	139
Given a copy of my treatment plan	64; 46.4%	14; 10.1%	14; 10.1%	24; 17.4%	22; 15.94%	138
Encouraged to go to a specific group or class to help me cope with my chronic condition	80; 56.3%	15; 10.6%	24; 16.9%	13; 9.2%	10; 7.0%	142
Asked questions, either directly or on a survey, about my health habits	62; 44.0%	24; 17.0%	24; 17.0%	18; 12.8%	13; 9.2%	141
Sure that my doctor or nurse thought about my values, beliefs, and traditions when they recommended treatments to me	36; 25.9%	19; 13.7%	13; 9.4%	44; 31.7%	27; 19.4%	139
Helped to make a treatment plan that I could carry out in my daily life	39; 28.3%	19; 13.8%	23; 16.7%	32; 23.2%	25; 18.1%	138
Helped to plan ahead so I could take care of my condition even in hard times	51; 37.0%	23; 16.7%	15; 10.9%	31; 22.5%	18; 13.0%	138
Asked how my chronic condition affects my life	48; 34.8%	24; 17.4%	21; 15.2%	23; 16.7%	22; 15.9%	138
Contacted after a visit to see how things were going	101; 73.2%	21; 15.2%	5; 3.6%	9; 6.5%	2; 1.4%	138
Encouraged to attend programs in the community that could help me	84; 61.3%	25; 18.3%	15; 101.0%	9; 6.6%	4; 2.9%	137
Referred to a dietitian, health educator or counsellor	80; 58.8%	19; 14.0%	20; 14.7%	8; 5.9%	9; 6.6%	136
Told how my visits with other types of doctors, like an eye doctor or other specialist, helped my treatment	69; 50.0%	21; 15.2%	22; 15.9%	13; 9.4%	13; 9.4%	138
Asked how my visits with other doctors were going	53; 38.4%	27; 19.6%	19; 13.8%	21; 15.2%	18; 13.0%	138

**Table 4:** Response to the request to complete the statement: “*Over the past 6 months, when I received care for my cancer, I was…*”

	None of the time (*n*; %)	A little of the time (*n*; %)	Some of the time (*n*; %)	Most of the time (*n*; %)	Always (*n*; %)	Total (*n*)
Asked for my ideas when we made a treatment plan	51; 39.5%	25; 19.4%	17; 13.2%	18; 14.0%	18; 14.0%	129
Given choices about treatment to think about	44; 34.1%	27; 20.9%	18; 14.0%	23; 17.8%	17; 13.1%	129
Asked to talk about any problems with my medicines or their effects	44; 34.1%	21; 16.3%	16; 12.4%	20; 15.5%	28; 21.7%	129
Given a written list of things I should do to improve my health	89; 69.0%	12; 9.3%	14; 10.9%	8; 6.2%	6; 4.7%	129
Satisfied that my care was well organized	26; 20.0%	20; 15.4%	19; 14.6%	36; 27.7%	29; 22.3%	130
Shown how what I did to take care of myself influenced my condition	58; 45.0%	20; 15.5%	29; 14.7%	29; 14.7%	23; 10.1%	129
Asked about my goals in caring for my condition	72; 55.8%	13; 10.1%	20; 15.5%	16; 12.4%	8; 6.2%	129
Helped to set specific goals to improve my eating and exercise	78; 60.9%	22; 17.2%	13; 10.2%	7; 5.5%	8; 6.3%	128
Given a copy of my treatment plan	82; 64.1%	16; 12.5%	11; 8.6%	9; 7.0%	10; 7.8%	128
Encouraged to go to a specific group or class to help me cope with my chronic condition	87; 67.4%	16; 12.4%	14; 10.9%	7; 5.4%	5; 3.9%	129
Asked questions, either directly or on a survey, about my health habits	73; 56.6%	19; 14.7%	16; 12.4%	12; 9.3%	9; 7.0%	129
Sure that my doctor or nurse thought about my values, beliefs, and traditions when they recommended treatments to me	39; 30.7%	20; 15.8%	14; 11.0%	25; 19.7%	29; 22.8%	127
Helped to make a treatment plan that I could carry out in my daily life	49; 38.6%	18; 14.2%	15; 11.8%	25; 19.7%	20; 15.8%	127
Helped to plan ahead so I could take care of my condition even in hard times	56; 44.4%	19; 15.1%	19; 15.1%	21; 16.7%	11; 8.7%	126
Asked how my chronic condition affects my life	58; 46.0%	20; 15.9%	17; 13.5%	12; 9.5%	19; 15.1%	126
Contacted after a visit to see how things were going	9; 76.2%	8; 6.4%	12; 9.5%	6; 4.8%	4; 63.2%	126
Encouraged to attend programs in the community that could help me	89; 70.1%	15; 11.8%	12; 9.5%	6; 4.7%	5; 3.9%	127
Referred to a dietitian, health educator or counsellor	92; 73.0%	13; 10.3%	8; 6.4%	9; 7.1%	4; 3.2%	126
Told how my visits with other types of doctors, like an eye doctor or other specialist, helped my treatment	81; 63.3%	15; 11.7%	12; 9.4%	12; 9.4%	8; 6.3%	128
Asked how my visits with other doctors were going	60; 46.89%	24; 18.8%	15; 11.7%	13; 10.1%	16; 12.5%	128

**Table 5:** PACIC overall score and subscales

	Chronic disease	Cancer	Difference	*p*^1^	*p*^2^
	Mean (SD)	Mean (SD)	Mean [95% CI]		
Activation, *n* = 128	2.8 (1.3)	2.6 (1.4)	0.2 [0.02, 0.5]	0.03	0.0497
Delivery, *n* = 129	2.7 (1.1)	2.4 (1.1)	0.3 [0.1, 0.5]	0.001	< 0.001
Goal, *n* = 128	2.2 (1.1)	1.9 (1.0)	0.4 [0.2, 0.5]	< 0.001	< 0.001
Counselling, *n* = 127	2.8 (1.3)	2.5 (1.3)	0.2 [0.04, 0.4]	0.02	0.003
Follow-up, *n* = 128	1.9 (0.9)	1.8 (1.0)	0.2 [0.03, 0.3]	0.02	0.008
Overall, *n* = 130	2.4 (1.0)	2.2 (1.0)	0.3 [0.1, 0.4]	< 0.001	< 0.001

^1^*p* values are derived from paired sample *t*-test; ^2^*p* values are derived from Wilcoxon matched-pairs signed-ranks test

## Discussion

This cross-sectional survey of Australian breast cancer survivors highlights that comorbid chronic conditions were common in this group; their presence correlated with inferior perceived health, and their care poorly aligned with best practice in chronic condition management. Nearly 90% of respondents had some form of chronic condition in addition to cancer and nearly a quarter had four or more. These rates were higher than some of the other studies of breast cancer survivors likely reflecting the selection bias of survey respondents. In addition, our study considered not only the conditions listed explicitly by the CCI but also those that the CCI would normally categorise as “other” but are common among breast cancer survivors such as lymphoedema, neuropathy, osteoporosis or arthritis [20–23]. This highlights the relative limitations of the CCI in this population and the need for developing comorbidity assessment tools that are specific for the types of comorbidities that are more likely to occur in women with breast cancer.

The assessment of the quality of care received for the management of chronic conditions demonstrated poor alignment with best practice in chronic condition management in this otherwise relatively young, healthy, insured and at least e-health literate population, given the mode of distribution of the survey. One could argue that a potential explanation might be the lack of awareness or prioritisation of management of chronic conditions by the participants themselves. However, this possibility seems unlikely given that the observed quality of cancer care in this cohort was even worse. This observation suggests the presence of more systemic deficiencies in the care delivery for cancer survivors or perhaps in chronic care delivery in general. PACIC, the tool used in the present study, is designed to assess the delivery of chronic care management from the patient’s perspective and has been extensively used in other chronic conditions [24]; but we are not aware of similar data in cancer. Further research into the quality of cancer care, and specifically the care of comorbid chronic conditions in the context of cancer care, is warranted.

The study findings need to be interpreted in the context of the survey limitations. The response rate was low, consistent with this type of survey, but likely to lead to a significant selection bias. It is notable however that respondents were relatively young, considered themselves healthy and with better health literacy given the mode of recruitment. It is therefore possible that the findings in this study underestimate the problem of comorbidities. Comorbid chronic conditions are more likely to occur in patients who are older, frailer and in those with lower socioeconomic status where both cancer outcomes and outcomes of comorbidities are poor [25]. Future studies should focus on experiences of living with chronic disease specifically in these populations. If these findings are replicated in other studies with larger response rates, more consideration could be given to models of care based on the chronic care model [26]. Further consideration could be made of training of primary care providers and cancer care providers in chronic condition management and the role of self-management to improve outcomes for patients living with cancer and comorbid chronic conditions.

In conclusion, comorbid chronic conditions are common among breast cancer survivors. In this population of survivors with good health status and high rates of private insurance, the management of chronic conditions and the management of breast cancer itself demonstrated limited alignment with established chronic disease management principles. This indicates important gaps in care delivery as well as missed opportunities for early intervention that warrant further attention.

## Supplementary Information

Below is the link to the electronic supplementary material.

Supplementary file1 (DOCX 940 KB)
