# Supplementary material for: Comorbidities and their management in women with breast cancer—an Australian survey of breast cancer survivors
Source: Support Care Cancer. 2023 Mar 14;31(4):212. doi: 10.1007/s00520-023-07678-7 (PMC10014784; doi:10.1007/s00520-023-07678-7)
Supplement: Supplementary file 1 — Supplementary file1 (DOCX 940 KB) [file 520_2023_7678_MOESM1_ESM.docx]

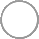

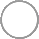


# 1. Welcome to Improving Management of Chronic Conditions in Cancer Survivors Survey

## You are invited to take part in the study about improving management of chronic conditions in cancer survivors. The main aim of this study is to improve understanding of the health burden of chronic conditions in breast cancer survivors and to identify strategies to improve management of these conditions.

**The following survey is aimed at breast cancer survivors with other chronic conditions and in it you will find a number of questions about your health and health management. Please answer the questions as honestly as you can. If you find that any particular question is too sensitive, you do not need to answer it. Your answers will remain strictly confidential. When you have finished the survey please click ‘Submit survey’.**

**If you prefer to complete a hard copy of the questionnaire and post it you can print it, complete it and post it to Ms Suzana Freegard, Level 4 Room 100, Flinders Centre for Innovation in Cancer, Flinders Drive, Bedford Park SA 5042. Alternatively, you can contact Suzana on (08) 8404 2830 or on** [**suzana.freegard@flinders.edu.au**](mailto:suzana.freegard@flinders.edu.au) **and she will send you a copy and self-addressed envelope to post the questionnaire to us.**

**This study has been approved by the Southern Adelaide Clinical Human Research Ethics Committee. If you have any questions about the questionnaire, you can contact Ms Suzana Freegard on (08) 8404 2830 or on** [**suzana.freegard@flinders.edu.au**](mailto:suzana.freegard@flinders.edu.au)

### 1. Are you happy to proceed?

Yes No


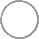

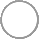

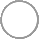

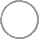

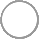

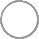

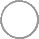

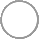

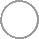

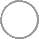

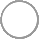

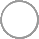

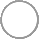

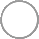

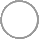

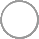

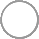

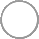


# 2. About you

### What is your age?

1. What is your marital status?

Single Married De facto Divorced Widowed

Prefer not to tell

### What is your employment status?

Employed Unemployed Retired Home duties Other

Prefer not to tell

### What is your average gross annual household income?

$0 - $6,000

$6,000 - $35,000

$35,000 - $80,000

$80,000 - $180,000

Over $180,000 Prefer not to tell


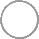

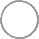

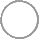

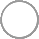

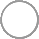

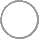


# 3. About you

### What is your living situation?

Living alone Other

Prefer not to tell

### Do you currently have private health insurance?

Yes, I do No, I do not

Prefer not to tell

### With what culture/group do you identify? (please tick all that apply)

Australian

Other Caucasian Aboriginal

Torres Strait Islander Asian

African

Latin American Other

Prefer not to tell

# 4. About your chronic conditions

### 9. Which chronic conditions, aside from a history of breast cancer, do you currently have? (check all that apply)

Myocardial Infraction Congestive Heart Failure Peripheral Vascular Disease Cerebrovascular Disease

Chronic Obstructive Pulmonary Disease (COPD) Asthma

Peptic Ulcer Disease Liver Disease - mild

Liver Disease with end-organ damage (moderate to severe) Moderate to severe Chronic Kidney Disease

Diabetes Mellitus - uncomplicated

Diabetes Mellitus with end-organ damage (moderate to severe) Arm and/or leg weakness

Leukemia

Malignant Tumor - not metastatic Malignant Tumor - metastatic Dementia

Mental Disorders (including depression and bipolar disorder) AIDS

Obesity Chronic pain

Persistent fatigue Peripheral Neuropathy

Any other type of cancer or chronic condition (please specify):


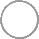

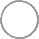

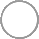

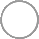

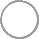

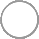

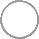

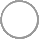

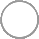

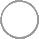

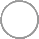

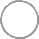

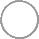

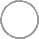

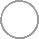

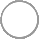

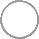

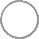

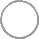

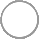

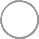

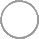

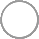

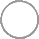

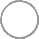

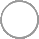

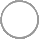

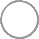

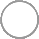

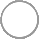

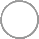

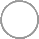

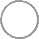

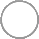

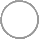

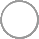

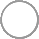

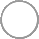

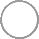

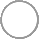


# 5. Health and physical function

### In general would you say your health is excellent, very good, good, fair or poor?

Excellent Very good Good

Fair Poor

### How much difficulty, on average, do you have with the following physical activities:

No difficulty A little difficulty Some difficulty A lot of difficulty Unable to do

Stooping, crouching or kneeling?

Lifting, or carrying objects as heavy as 10 ponds?

Reaching or extending arms above shoulder level?

Writing, or handling and grasping small objects?

Walking a quarter of a mile?

Heavy housework such as scrubbing floors or washing windows?

### Because of your health or a physical condition, do you have any difficulty shopping for personal items (like toilet items or medicines)?

Yes, but I get help with shopping for personal items

Yes and I do not get help with shopping for personal items

No, I do not have any difficulty with shopping for personal items I don't shop for personal items because of my health

I don't shop for personal items for other reasons


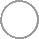

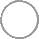

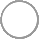

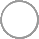

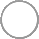

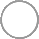

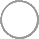

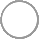

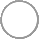

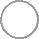

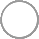

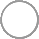

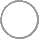

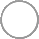

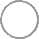

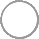

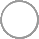

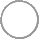

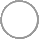

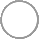


# 6. Health and physical function

### Because of your health or a physical condition, do you have any difficulty managing money (like keeping track of expenses or paying bills)?

Yes, but I get help with managing money

Yes and I do not get help with managing money

No, I do not have any difficulty with managing money I don't manage money because of my health

I don't manage money for other reasons

### Because of your health or a physical condition, do you have any difficulty walking across the room? (USE OF CANE OR WALKER IS OK)

Yes, but I get help with walking across the room

Yes and I do not get help with walking across the room No, I do not have any difficulty walking across the room I don't walk across the room because of my health

I don't walk across the room for other reasons

### Because of your health or a physical condition, do you have any difficulty doing light housework (like washing dishes, straightening up, or light cleaning)?

Yes, but I get help with doing light housework

Yes and I do not get help with doing the light housework No, I do not have any difficulty doing light housework

I don't do light housework because of my health I don't do light housework for other reasons

### Because of your health or physical condition, do you have any difficulty bathing or showering?

Yes, but I get help with bathing or showering

Yes and I do not get help with bathing or showering

No, I do not have any difficulty with bathing or showering I don't bath or shower because of my health

I don't bath or shower for other reasons


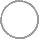

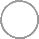

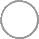

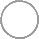

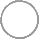

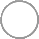

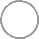

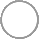

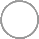

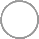

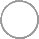

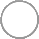

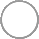

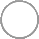


# 7. Chronic condition care

## Staying healthy can be difficult when you have a chronic condition. We would like to learn about the type of help with your condition you get from your health care team. This might include your regular doctor, his or her nurse, or physician’s assistant who treats your illness. Your answers will be kept confidential and will not be shared with your physician or clinic. We will ask you these questions with regards to the care you received for cancer and the care you received for other conditions separately.

### 17. Over the past 6 months, when I received care for my chronic conditionsaside from cancer, I was:

None of the time A little of the time Some of the time Most of the time Always

Asked for my ideas when we made a treatment plan.

Given choices about treatment to think about.

Asked to talk about any problems with my medicines or their effects.

Given a written list of things I should do to improve my health.

Satisfied that my care was well organized.

Shown how what I did to take care of myself influenced my condition.

Asked about my goals in caring for my condition.

Helped to set specific goals to improve my eating and exercise.

Given a copy of my treatment plan.

Encouraged to go to a specific group or class to help me cope with my chronic condition.

Asked questions, either directly or on a survey, about my health habits.

# 8. Chronic condition care

### 18. Over the past 6 months, when I received care for my chronic conditionsaside from cancer, I was:

None of the time A little of the time Some of the time Most of the time Always

Sure that my doctor or nurse thought about my values, beliefs, and traditions when they recommended treatments to me.

Helped to make a treatment plan that I could carry out in my daily life.

Helped to plan ahead so I could take care of my condition even in hard times.

Asked how my chronic condition affects my life.

Contacted after a visit to see how things were going.

Encouraged to attend programs in the community that could help me.

Referred to a dietitian, health educator or counselor.

Told how my visits with other types of doctors, like an eye doctor or other specialist, helped my treatment.

Asked how my visits with other doctors were going.

# 9. Cancer-related care

### 19. Over the past 6 months, when I receivedcancer-related care, I was:

None of the time A little of the time Some of the time Most of the time Always

Asked for my ideas when we made a treatment plan.

Given choices about treatment to think about.

Asked to talk about any problems with my medicines or their effects.

Given a written list of things I should do to improve my health.

Satisfied that my care was well organized.

Shown how what I did to take care of myself influenced my condition.

Asked about my goals in caring for my condition.

Helped to set specific goals to improve my eating and exercise.

Given a copy of my treatment plan.

Encouraged to go to a specific group or class to help me cope with my chronic condition.

Asked questions, either directly or on a survey, about my health habits.

# 10. Cancer-related care

### 20. Over the past 6 months, when I receivedcancer-related care, I was:

None of the time A little of the time Some of the time Most of the time Always

Sure that my doctor or nurse thought about my values, beliefs, and traditions when they recommended treatments to me.

Helped to make a treatment plan that I could carry out in my daily life.

Helped to plan ahead so I could take care of my condition even in hard times.

Asked how my chronic condition affects my life.

Contacted after a visit to see how things were going.

Encouraged to attend programs in the community that could help me.

Referred to a dietitian, health educator or counselor.

Told how my visits with other types of doctors, like an eye doctor or other specialist, helped my treatment.

Asked how my visits with other doctors were going.

# 11. End of survey

### Is there anything you wish to add?

No Yes

**Thank you for completing this survey. Your participation is very much appreciated.**

**Would you be willing to speak to our research team about your experiences? We would like to interview approximately 20 women to gain greater understanding about their experiences of living with cancer and chronic conditions. If you are interested in taking part, we will provide you with further information.**

### Please indicate if you are interested in being contacted about participating in a telephone interview.

No, I do not wish to be contacted about taking part in a telephone interview. Yes, I can be contacted about taking part in a telephone interview .

If yes, please provide your name and preferred contact details (phone number or email address):

**You have now concluded your participation. Thank you!**
